# Direct cost comparison of minimally invasive punch technique versus traditional approaches for percutaneous bone anchored hearing devices

**DOI:** 10.1186/s40463-017-0222-2

**Published:** 2017-06-12

**Authors:** Yaeesh Sardiwalla, Nicholas Jufas, David P. Morris

**Affiliations:** 1 Faculty of Medicine, Dalhousie University, Halifax, NS Canada; 2 Division of Otolaryngology – Head and Neck Surgery, Dalhousie University, Halifax, NS Canada; 3 Discipline of Surgery, Sydney Medical School, University of Sydney, Sydney, Australia; 4 QEII Health Science Center - VG Site Otolaryngology, 5820 University Ave - Rm 3037, Halifax, NS B3H 2Y9 Canada

**Keywords:** Costs and cost analysis, Otologic surgical procedures, Bone conduction, Minimally invasive surgical procedures

## Abstract

**Background:**

Minimally Invasive Ponto Surgery (MIPS) was recently described as a new technique to facilitate the placement of percutaneous bone anchored hearing devices.

The procedure has resulted in a simplification of the surgical steps and a dramatic reduction in surgical time while maintaining excellent patient outcomes. Given these developments, our group sought to move the procedure from the main operating suite where they have traditionally been performed. This study aims to test the null hypothesis that MIPS and open approaches have the same direct costs for the implantation of percutaneous bone anchored hearing devices in a Canadian public hospital setting.

**Methods:**

A retrospective direct cost comparison of MIPS and open approaches for the implantation of bone conduction implants was conducted. Indirect and future costs were not included in the fiscal analysis.

A simple cost comparison of the two approaches was made considering time, staff and equipment needs. All 12 operations were performed on adult patients from 2013 to 2016 by the same surgeon at a single hospital site.

**Results:**

MIPS has a total mean reduction in cost of CAD$456.83 per operation from the hospital perspective when compared to open approaches. The average duration of the MIPS operation was 7 min, which is on average 61 min shorter compared with open approaches.

**Conclusion:**

The MIPS technique was more cost effective than traditional open approaches. This primarily reflects a direct consequence of a reduction in surgical time, with further contributions from reduced staffing and equipment costs. This simple, quick intervention proved to be feasible when performed outside the main operating room. A blister pack of required equipment could prove convenient and further reduce costs.

## Background

Percutaneous bone-anchored hearing devices (BAHD) or bone conduction hearing implants (BCHI) rely on a secure osseointegrated implant first described in the 1970s by Prof. Brånemark [1]. The first BAHD was placed in 1977 by Anders Tjellstrom [1]. The product has found application in the rehabilitation of single sided deafness (SSD) and a range of hearing losses where there is an intolerance of or an inability to wear conventional amplification [2]. BAHD transmit sound directly through the temporal bone to the inner ear. The three main components are the osseointegrated screw or fixture, the skin penetrating abutment and the removable sound processor that connects externally to the abutment [3]. The implantation of the device has been shown to be safe in both adults and children with many publications demonstrating beneficial effects on hearing [2, 4].

A number of open techniques have been described where the drilling stages of the surgery are performed under direct vision. Traditional approaches have varied in the style of initial incision but most of them involved significant soft tissue undermining and excision in order to obtain the thin, hairless and immobile implant site that was deemed optimal for long term stability [5]. Such techniques were time-consuming and were often associated with significant bleeding. Many were previously performed under general anaesthesia at our institution for precisely these reasons.

In more recent years there has been a move to less soft tissue reduction and simplified linear incisions that in turn demanded the use of longer abutments [6]. As the surgical technique has become less invasive and surgical times have reduced, the procedures have been exclusively performed under local anaesthetic in our operating room.

In 2011, Hultcrantz et al. described the Minimally Invasive Ponto Surgery (MIPS) procedure using a 5 mm dermal punch to remove the limited tract of soft tissue needed to accommodate the Ponto (Oticon, Copenhagen, Denmark) abutment [7]. The drilling procedure was then completed in seconds, through a cannula placed to protect the skin and soft tissues while holding cooling fluid. MIPS heralds a departure from the traditional “open approach” to percutaneous fixture placement. Soft tissue preservation and longer abutments placed in a few simple surgical steps mark a natural evolution in technique where technical simplification appears possible without compromise to patient care. Early evidence suggests that techniques preserving soft tissue result in favorable outcomes [5].

In our series, we saw the significant reduction in surgical time and procedural invasiveness as a logistical opportunity to move such cases out of the main operating room. Our motivation to do so was driven by our desire to reduce the impact of this surgical intervention on our patients while maintaining a high standard of care and safety. As medical professionals we are constantly striving to deliver high quality, patient-centered care, balancing optimal outcomes from our interventions with the efficient use of finite resources [8].

Although direct, indirect and future costs should be considered to fully evaluate the monetary value of a health care intervention, this study focuses on direct costs. Direct costs include those of the surgeon, anesthesiologist, nursing staff, hospital resources and equipment costs. The objective of this study was to conduct a direct cost analysis comparing MIPS performed outside the OR to the traditional more open techniques in an OR setting at the Queen Elizabeth II Health Sciences Centre in Halifax, Nova Scotia. This is the first cost analysis of this type to be reported.

## Methods

### Patient information

A cost difference analysis of open approaches and MIPS procedures was performed using a retrospective analysis of direct costs. A total of 12 adult patients operated on by a single surgeon as day case procedures were evaluated. Indirect and future costs were excluded. There were 6 patients who received an abutment implant prior to July 2015 using the open approach in the OR setting. This sample was compared 6 MIPS procedures performed outside of the OR under local anesthetic. A minor procedures room (brachytherapy suite) that conformed to Infection Prevention and Control standards, particularly that of adequate air exchange, was used for the operation during this transition. A convenience sampling approach was used for patients in each group.

### Cost analysis

A cost difference approach was used to evaluate the total costs of each procedure. The relevant resources to be evaluated were identified by mapping the patient’s health journey. Costs that were similar between open approaches and MIPS procedures were negated in the analysis. The negated costs included pre-operative workup and consultation, hospital admission, as well as post-operative follow up (see Discussion for clarification). As a result, only costs associated with the operative procedure itself were included in this direct cost analysis.

The costs and resources evaluated in this study were broken down in four major groups:

1. Surgeon’s fee
2. Nurses’ fees
3. Anesthesiologist’s fee
4. Operative set-up/equipment

Costs associated with the two procedures were obtained from the Queen Elizabeth II Health Sciences Centre Business Department and relevant nursing staff following the guidance of previously published studies from our institution [9]. The average hourly salary for the health care providers involved in the operations was calculated. Billing codes through Medical Services Insurance (MSI) were used to determine the annual salary of surgeons, and divided by the average number of hours worked each week to yield an average hourly salary [9]. The cost of sterilization and preparation of equipment trays for each procedure were itemized and priced by the business department. The costs of the anaesthetic items were not able to be included in the calculations due to limitations at our institution.

All costs were reported in Canadian Dollars (CAD).

### Procedure mean time

For open approaches, OR records were sourced and the operating time was determined by subtracting the end time (patient leaving OR) from the start time (first incision). The MIPS time was calculated prospectively from the moment of skin punch, to the moment the healing cap had been placed.

## Results

The mean time for the MIPS procedure was 5 min and 55 s (0.10 h) and 1 h and 7 min (1.13 h) for the open approaches. The cost of staffing was $44.72 per hour for nursing, $140.00 per hour for the surgeons and $125.00 per hour for the anesthesiologist at our facility. Two additional nurses and a single anesthesiologist were required for the open approaches in the OR versus MIPS. Total mean intraoperative costs for the open approach were $451.05 and $18.22 for MIPS. The MIPS technique therefore produced a cost saving of $432.83 compared to the open approaches in terms of provider cost. Intraoperative costs are summarized in Table 1.

**Table 1:** The costs and number of healthcare providers (HCP) required for MIPS versus open techniques

Procedure type	HCP	Number of HCP	Hourly wage ($/h)	Hours	Total cost ($)
MIPS	Nurse	1	44.72	0.10	4.41
	Surgeon	1	140.00	0.10	13.81
				Total Provider Cost	18.22
Open/Incision	Nurse	3	44.72	1.13	151.60
	Surgeon	1	140.00	1.13	158.20
	Anaesthesiologist	1	125.00	1.13	141.25
				Total Provider Cost	451.05

The total cost for sterilization of the equipment tray used in the MIPS procedure was found to be $24.00 less than the standard open approaches. Costs of disposables and anesthesia resources were not available for our facility and therefore not included.

The MIPS technique produced a $456.83 total cost saving when compared to open approaches for insertion of percutaneous bone anchored hearing devices. The cost saving can be broken down into the equipment and provider costs.

## Discussion

As clinicians we are primarily driven by safety and quality of care measures when faced with the choice of a new intervention for our patients. Once satisfied that these have been demonstrated, logistical issues of cost become our next responsible consideration. MIPS offers the opportunity of cost saving with no suggested negative impact to patient care [5, 7].

We confirmed a total cost saving of $456.83 for MIPS compared the open approaches. The comparative cost saving calculated is independent of surgical venue meaning that the cost saving will be evident at institutions where moving MIPS outside the main OR is not possible. Our attempts to quantify costs associated with the preparation and sterilization of the MIPS equipment tray was somewhat challenging. We were only able to show a small cost saving of $24.00 for equipment costs as bulk sterilization in our centre that has both large turnover and capacity is likely to under-estimate the potential savings that exist in a smaller facility or office setting.

**Fig. 1:**
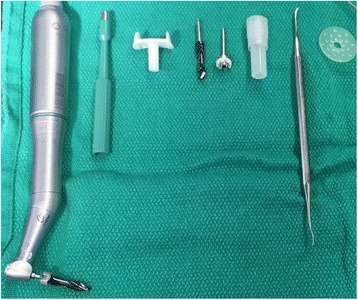
Proposed reduction in surgical tray. From *left*: Drill with bit, skin punch, cannula, countersink drill bit, handpiece connector, combined abutment and fixture, raspatorium/ dissector, healing cap

It is intuitive and entirely feasible that equipment costs can be reduced even further in the future. Once out of the operating room setting, we reviewed the items required for this short procedure. The arrangement shown below (Fig. 1) is a significant departure from the previous requirement to open a full surgical tray. When soft tissue reduction was required, bleeding was the rule not the exception and cautery was essential. If the small dissector could be made disposable it could be included with a skin punch as additions to the currently offered blister-packed set. In short, the drill would be the only piece of equipment requiring sterilization. These proposals have clear implications both for costs associated with initial outlay to purchase items and equipment and for the subsequent costs of sterilizing and maintaining such equipment.

Small incidental costs that are no greater than those associated with the traditional approach include limited hair-shave, infiltration of local anaesthetic (at which stage skin thickness can be estimated) and sterile preparation and drape. A syringe with an attached plastic cannula and a bottle of cold saline are required for irrigation. Ribbon gauze with ointment can be left to the surgeon’s discretion or a sponge disc could be a further addition to the blister pack.

In moving MIPS out the main suite of operating rooms, continued patient safety was our top priority. In close consultation with the infection control teams at our institution, the MIPS procedures were performed in a treatment room that still met particular specifications. Importance was placed on adequacy of air exchange. All supplies were removed from the room, appropriate Personal Protective Equipment (PPE) was worn, there was an external sink for surgical scrub, the door was not opened during the procedure and a terminal clean was performed between cases.

**Fig. 2:**
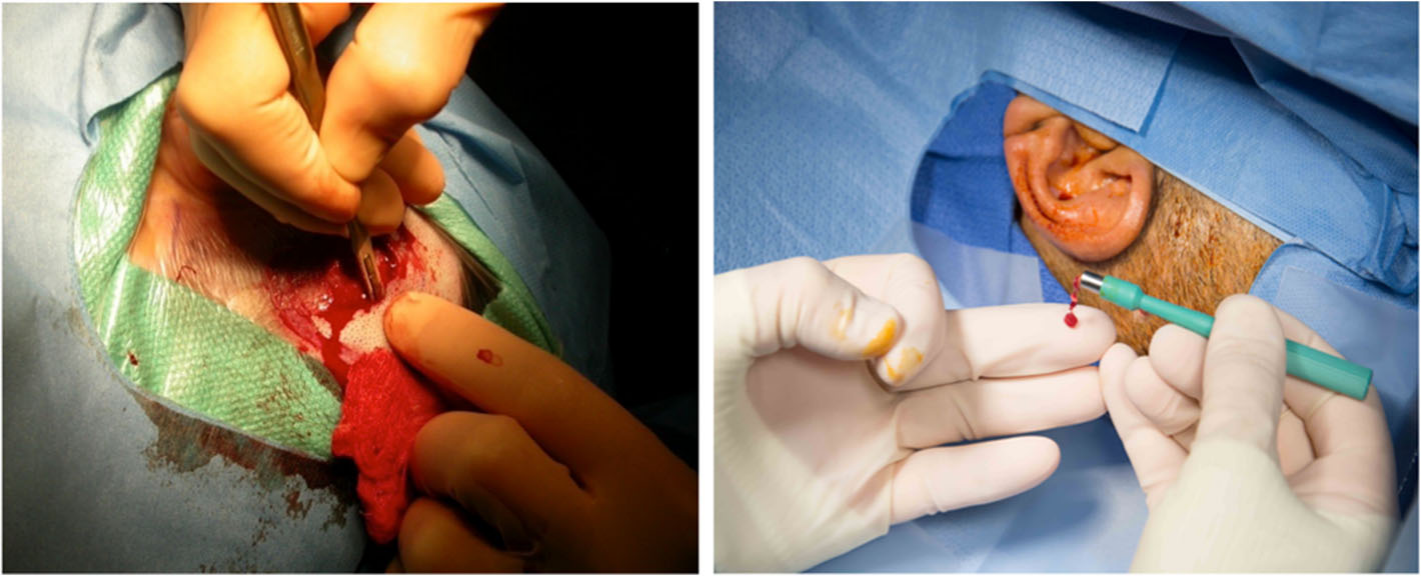
Bleeding and dissection are strikingly reduced with the MIPS approach (*right*) compared to the more intrusive open approach (*left*)

There are theoretical concerns regarding any procedure where dura, brain tissue or cerebrospinal fluid (CSF) might be encountered and equally so where surgical procedures might generate a blood and bone dust aerosol. There is precedent for procedures such as burr hole drilling for intracranial pressure monitoring being performed at the bed side with proven safety [10]. We propose that surgical venue is not the most important factor in determining operative risk, provided that correct protocols are followed, and sterility concerns are satisfied. There have been no long-term complications such as fixture failures in our cohort and all devices are still being worn by patients. Our group will be publishing a case-series evaluation of this cohort and long-term data of MIPS outcomes from other groups will soon be made available [11, 12]. Institutionalized memory of earlier more bloody techniques used for implant placement may need to be faced and discussed during such a transition (Fig. 2).

This study employed a simple cost difference analysis only taking into account measurable direct costs. The simplicity of this approach made the cost calculations easy to compute. The only direct cost that was not captured was that of anaesthetic resources during the operation due to facility constraints except for the anesthesiologist’s fee. These would be expected to be greater in the open approach not least as many such cases are performed using general anaesthetic requiring intubation, monitoring and recovery. The cost saving of $456.83 could therefore grossly underestimate the total direct cost saving.

An inherent shortcoming of a direct cost analysis is that it fails to capture future and indirect costs. Indirect costs could be categorized as those borne by the patient or those assumed by the health service. Reduced aftercare is likely to represent a further potential cost saving. In our experiences so far, MIPS has offered a faster recovery time as there is little to heal and this is consistent with results that involved less soft tissue undermining [5].

Indirect cost savings to the healthcare system include the benefit of regaining over two hours of prime operating time including procedure and turnaround that frees up facilities and staff to allow for other more urgent operations to be completed. In addition to this, the logistical consideration of performing MIPS procedures as day cases is that a hospital bed is not required. Procedures will not be cancelled due to a lack of overnight hospital bed availability. Hospital resources can be channeled more efficiently for surgical procedures that necessitate an overnight bed. Follow-up studies examining patient satisfaction, recovery time, quality of life, medical outcomes and functionality have not yet been published but are much anticipated and currently ongoing [11, 12].

A limitation of this study relates to imperfect case matching between MIPS and open approach surgeries. Patient demographics and patient comorbidities likely affect surgical duration, and this was not strictly controlled for in our study, but randomization was likely with our convenience sampling approach. Larger patient numbers, matching for measurable characteristics or systematic randomization in a prospective study could mitigate the potential for bias in future analysis. Despite this, there is a clear and intuitive trend of the opportunity for cost saving.

As medical professionals, we have a responsibility to ensure that the services we provide are safe, high quality and economically efficient. When alternative procedures to the current standards have been demonstrated to show those qualities, they should be considered as changes to normal practice. This study has demonstrated the obvious economic benefit of taking the simplified MIPS technique out of the main operating suite. We have confirmed that MIPS has a reduced mean time of operation and staff costs. Equipment cost savings have not been fully captured in this study and are likely to have been under-estimated.

## Conclusion

This is the first published study documenting the direct cost benefits of the MIPS procedure compared to open approaches. Lower equipment costs and reduced healthcare professional fees make this true regardless of where the procedure is performed. The study has also demonstrated the suitability of performing MIPS outside the traditional operating room setting, as it is quicker and less invasive. In moving MIPS out of our main OR suite we were able to better utilize facilities and staff to allow for other more pressing operations to be completed. This study has calculated a considerable direct cost saving of at least $456.83 per operation. This figure likely grossly underestimates the true total cost saving to the health care system and the individual patient when all direct, indirect and future costs are considered.

## Abbreviations

BAHD: Bone anchored hearing devices
BCHI: Bone conduction hearing implants
CAD: Canadian Dollars
CSF: Cerebrospinal fluid
HCP: Healthcare providers
MIPS: Minimally Invasive Ponto Surgery
MSI: Medical Services Insurance
OR: Operating room
PPE: Personal Protective Equipment
